# DRBD18 acts as a transcript-specific RNA editing auxiliary factor in *Trypanosoma brucei*

**DOI:** 10.1261/rna.080295.124

**Published:** 2025-02

**Authors:** Parul Pandey, Katherine Wackowski, Ashutosh P. Dubey, Laurie K. Read

**Affiliations:** Department of Microbiology and Immunology, University at Buffalo Jacobs School of Medicine and Biomedical Sciences, Buffalo, New York 14203, USA

**Keywords:** arginine methylation, posttranslational modification, RNA-binding protein, RNA editing, Trypanosome

## Abstract

Uridine insertion/deletion (U-indel) RNA editing of mitochondrial transcripts is a posttranscriptional modification in kinetoplastid organisms, resulting in the generation of mature mRNAs from cryptic precursors. This RNA editing process involves a multiprotein complex holoenzyme and multiple accessory factors. Recent investigations have highlighted the pivotal involvement of accessory RNA-binding proteins (RBPs) in modulating RNA editing in *Trypanosoma brucei*, often in a transcript-specific manner. DRBD18 is a multifunctional RBP that reportedly impacts the stability, processing, export, and translation of nuclear-encoded mRNAs. However, mass spectrometry studies report DRBD18–RESC interactions, prompting us to investigate its role in mitochondrial U-indel RNA editing. In this study, we demonstrate the specific and RNase-sensitive interaction of DRBD18 with multiple RESC factors. Depletion of DRBD18 through RNA interference in procyclic form *T. brucei* leads to a significant reduction in the levels of edited A6 and COIII mitochondrial transcripts, whereas its overexpression causes a notable increase in the abundance of these edited mRNAs. RNA immunoprecipitation/qRT-PCR analysis indicates a direct role for DRBD18 in A6 and COIII mRNA editing. We also examined the impact of arginine methylation of DRBD18 in the editing process, revealing that the hypomethylated form of DRBD18, rather than the arginine-methylated version, is essential for promoting these editing events. In conclusion, our findings demonstrate that DRBD18 directly affects the editing of A6 and COIII mRNAs, with its function being modulated by its arginine methylation status, marking the first report of a mitochondrial function for this protein and identifying it as a newly characterized RNA editing auxiliary factor.

## INTRODUCTION

The protozoan parasite, *Trypanosoma brucei*, is the causative agent of African sleeping sickness in humans; it also causes nagana in livestock (Pays et al. 2023). *T. brucei* undergoes a complex life cycle, transitioning between the mammalian host, where it resides in the bloodstream and numerous tissues, and the tsetse fly insect vector. *T. brucei* belongs to the order Kinetoplastida, which is notable for several unique biological characteristics (Lukeš et al. 2023). Kinetoplastids are so named due to the presence of a unique mitochondrial DNA called the kinetoplast or kDNA, usually arranged in the form of a network containing thousands of ∼0.5–10 kb-long minicircles that are heterogeneous in sequence and constitute the bulk of kDNA, and dozens of 20–40 kb-long maxicircles that resemble the mitochondrial genomes of other aerobic eukaryotes because they contain rRNA and protein-coding genes, mostly encoding subunits of the respiratory complexes (Shlomai 2004; Lukeš et al. 2005; Jensen and Englund 2012). In *T. brucei*, expression of 12 of the 18 maxicircle-encoded genes requires modification of mRNAs by extensive uridine insertion/deletion (U-indel) RNA editing to generate functional open reading frames (Read et al. 2016; Zimmer et al. 2018; Aphasizheva et al. 2020a). The key players in this process are 50–60 nt-long guide RNAs (gRNAs), which are predominantly encoded by minicircles. gRNAs direct the insertion and deletion of uridine residues through base-pairing interactions. The editing begins when the anchor region of the first gRNA associates with the 3′ never-edited region of the preedited mRNA, forming a short anchor duplex, while the rest of the gRNA forms an imperfect duplex with the mRNA. The editing machinery then modifies the mRNA according to the gRNA's coding region until gRNA and edited mRNA are fully complementary through Watson–Crick and G:U base-pairing, after which the first gRNA is removed by an unknown mechanism. The second gRNA then anchors to the previously created edited sequence. This process continues generally in the 3′ to 5′ direction until the mRNA is fully edited (Maslov and Simpson 1992).

U-indel editing is a coordinated process involving the association of RNA editing catalytic complexes (RECCs), the noncatalytic RNA editing substrate-binding complex (RESC), RNA editing helicase 2 complex (REH2C), and various auxiliary factors (Aphasizheva et al. 2020a). Three different RECCs catalyze endonuclease cleavage, U-indel, and RNA ligation (Carnes et al. 2011, 2017, 2022). RESC provides the platform for RNA editing and coordinates interactions between RECC, mRNA, and gRNA (Dubey et al. 2021; Liu et al. 2023; Wackowski et al. 2024). RESC consists of ∼20 proteins that are organized into dynamically interacting modules named guide RNA-binding complex (GRBC; a.k.a. MRB1 core or RESC-A) (Ammerman et al. 2012; Aphasizheva et al. 2014; Liu et al. 2023; Wackowski et al. 2024) and RNA editing mediator complexes (REMCs) (Aphasizheva et al. 2014, 2020a; Simpson et al. 2017). REH2C associates transiently with RECC or RESC and impacts editing specificity (Vondrušková et al. 2005; Kumar et al. 2016). Several other RNA-binding proteins (RBPs) have been reported as auxiliary factors that play a role in U-indel RNA editing of specific mRNAs and are associated with RESC and/or RECC. For example, kinetoplast mitochondrial RNA-binding proteins 1 and 2 (KMRP1 and 2) and KRBP16 have transcript-specific impacts on the editing of apocytochrome b (CYb) mRNA in insect midgut procyclic form (PF) *T. brucei* (Vondrušková et al. 2005; Fisk et al. 2009a; Tylec et al. 2019). Another RBP, KRGG1, is essential for the 3′ to 5′ progression of ATPase subunit 6 (A6) mRNA editing in mammalian bloodstream (BF) form of *T. brucei* (Carnes et al. 2023). The KREH1 RNA helicase modulates gRNA–mRNA interactions, substantially impacting initiator gRNAs (Dubey et al. 2023). Additional auxiliary factors are likely yet to be discovered that impact specific editing events throughout the *T. brucei* life cycle.

DRBD18 (Tb927.11.14090; double RNA-binding domain 18) is an RBP that regulates numerous aspects of nuclear-encoded gene expression, including stability, translation, nuclear export, and 3′ end processing (Lott et al. 2015; Mishra et al. 2021; Bishola Tshitenge et al. 2022; Ciganda et al. 2023; Bard et al. 2024). It is a critical factor in the maintenance of both BF and PF life cycle stages. In BF, DRBD18 is essential for keeping high levels of the pro-BF factor, RBP10 (Bishola Tshitenge et al. 2022). In PF, DRBD18 promotes the translation of PF-specific mRNAs and represses stumpy and metacyclic stage mRNAs (Ciganda et al. 2023; Bard et al. 2024). The latter effects are at least partially a result of DRBD18-regulated life cycle-specific poly(A) site selection, which maintains long 3′ UTRs, and presumably specific 3′-UTR elements, in PF (Bard et al. 2024). The molecular functions of DRBD18 in mRNA stabilization or destabilization and protein–protein interactions are markedly controlled by the methylation of arginine residues present between its two RNA-binding domains (Lott et al. 2015). Arginine methylation is a posttranslational modification that involves the transfer of a methyl group from the methyl donor *S*-adenosylmethionine to the terminal nitrogen of a peptidyl arginine (Bedford 2007). This posttranslational modification can affect both protein–protein and protein–nucleic acid interactions, with often profound effects on cellular physiology (Lorton and Shechter 2019). In keeping with the aforementioned DRBD18 functions, the protein is reportedly localized to both cytoplasm and nucleus (Lott et al. 2015; Bishola Tshitenge et al. 2022; Sunter et al. 2023). Interestingly, however, several mass spectrometry studies report interactions between DRBD18 and RESC (Lott et al. 2015; Aphasizheva et al. 2020b; Bishola Tshitenge et al. 2022; Liu et al. 2023), suggesting an additional role for DRBD18 in mitochondrial U-indel RNA editing.

In this study, we investigate the functional significance of DRBD18 in mitochondrial mRNA editing. We establish the specific and RNase-sensitive interaction between DRBD18 and numerous RESC factors. RNA interference (RNAi)-mediated depletion of DRBD18 in PF *T. brucei* results in a significant reduction in edited A6 (ATP synthase subunit 6) and COIII (cytochrome oxidase subunit III) mitochondrial transcripts; conversely, overexpression of DRBD18 leads to a notable increase in the same edited mRNAs. RNA immunoprecipitation (RIP)/qRT-PCR analysis supports a direct role for DRBD18 in A6 and COIII mRNA editing. Finally, mutagenesis analysis indicates that hypomethylated, but not methylmimic, DRBD18 is functional for the promotion of these editing events. Collectively, these findings demonstrate that the predominantly nuclear and cytoplasmic DRBD18 directly facilitates the editing of A6 and COIII transcripts in PF *T. brucei*, constituting the first report of a mitochondrial function for DRBD18 and highlighting it as a newly described RNA editing auxiliary factor.

## RESULTS

### DRBD18 interactions with RESC factors are specific and RNase-sensitive

**FIGURE 1.**
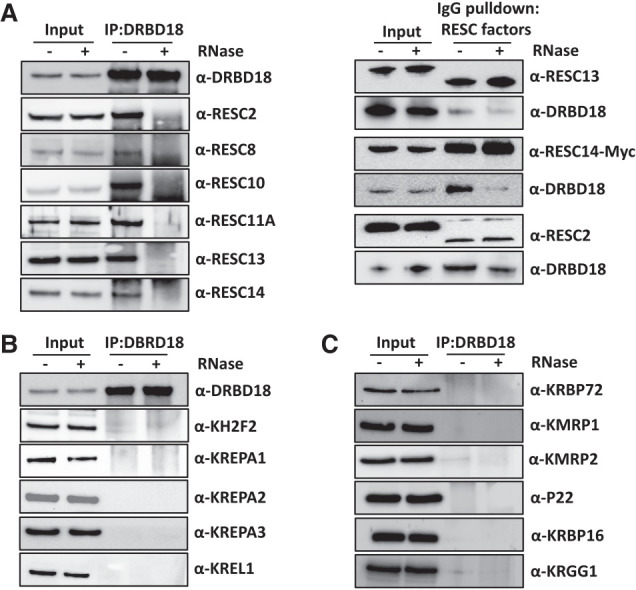
Interaction of DRBD18 with the editing machinery. (*A*) Interaction with RESC factors. (*Left*) DRBD18 was immunoprecipitated from mitochondrially enriched cell extracts that were treated with either RNase inhibitor (−RNase) or an RNase cocktail (+RNase) using α-DRBD18 antibody crosslinked protein A beads. Bound proteins were analyzed by western blot with native antibodies against RESC factors. (*Right*) RESC proteins (RESC13-MHT, RESC14-MHT, and RESC2-PTP) were precipitated from mitochondrially enriched cell extracts treated with either RNase inhibitor (−RNase) or an RNase cocktail (+RNase) using IgG beads. Target proteins were analyzed using native antibodies or α-Myc antibodies; associated DRBD18 was detected with α-DRBD18 antibodies. (*B*) As in *A*, *left*, but blotting for RNA editing holoenzyme components using native antibodies. (*C*) As in *A*, *left*, but blotting for RNA editing auxiliary factors using native antibodies. In *A* (*left*), *B*, and *C*, all pull-down lanes were loaded at 1000× input. *A* (*right*) was loaded as follows: For RESC13, DRBD18 was 1000× input and RESC13 was 200× input; for RESC14, both DRBD18 and RESC14 were 1000× input; for RESC2, DRBD18 was 1600× input and RESC2 was 100× input. The blots shown are representative of three biological replicates.

Mass spectrometry studies reported that DRBD18 interacts with multiple RESC factors (Lott et al. 2015; Aphasizheva et al. 2020b; Bishola Tshitenge et al. 2022; Liu et al. 2023). To gain broader insight into the associations between DRBD18 and components of the RNA editing machinery, we conducted co-immunoprecipitation (IP) experiments using available antibodies targeting RESC, RECC, REH2C, and auxiliary factors. IP of DRBD18 was performed using an α-DRBD18 antibody from mitochondria-enriched lysates treated either with an RNase cocktail or an RNase inhibitor, to determine RNase sensitivity of any interactions. We found that DRBD18 interacts with all tested RESC factors, specifically RESC2, RESC8, RESC10, RESC11A, RESC13, and RESC14, and these interactions are almost entirely RNase-sensitive (Fig. 1A, left). We confirmed the interactions with a subset of these proteins in the reverse direction by performing IgG pulldowns of RESC13-MHT (*M*yc-*H*is-*T*AP), RESC14-MHT, and RESC2-PTP (*P*rotC-*T*EV-*P*rotA) cell lines (Fig. 1A, right; Wackowski et al. 2024), and again established RNase-sensitive interactions between each of these proteins and DRBD18. We note that these DRBD18–RESC interactions may be direct or indirect through additional DRBD18 binding partners. We next interrogated DRBD18 association with non-RESC editing holoenzyme components. In contrast to RESC, we observed little to no interaction between DRBD18 and components of either REH2C (KH2F2) or RECC (KREPA1, KREPA2, KREPA3, and KREL1) (Fig. 1B). Similarly, DRBD18 exhibited little or no interaction with the RNA editing auxiliary factors, KRBP72, MRP1, MRP2, P22, KRBP16, or KRGG1 (Fig. 1C). Thus, DRBD18 specifically interacts with the RESC component of the RNA editing machinery in an RNase-sensitive manner.

### DRBD18 impacts the editing of A6 and COIII mitochondrial transcripts

**FIGURE 2.**
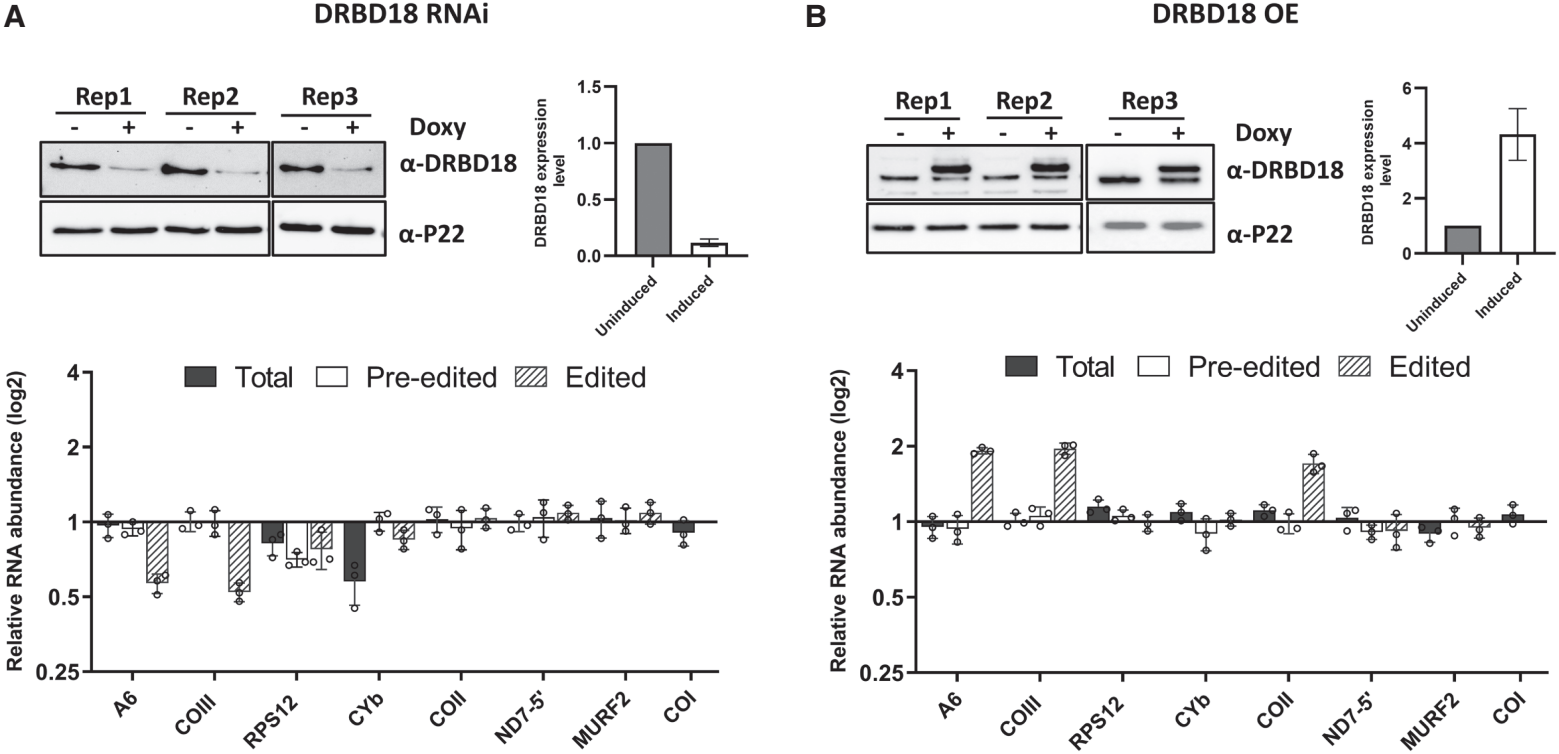
Effect of DRBD18 depletion and overexpression on U-indel RNA editing. (*A*, *top*) Western blot showing degree of DRBD18 knockdown in three biological replicate DRBD18 RNAi samples. Knockdown levels are quantitated on the *right*. (*Bottom*) qRT-PCR analysis of RNA isolated from uninduced (−Doxy) and induced (+Doxy) DRBD18 RNAi cells using primer sets designed to detect total, preedited, and edited mRNA of a subset of mitochondrial transcripts. The relative abundance of each transcript in induced versus uninduced RNAi cells is shown, and the RNA levels were normalized to 18S rRNA. Three biological replicates, each with three technical replicates, were performed. (*B*, *top*) Western blot showing degree of DRBD18 overexpression in three biological replicates. Levels of total DRBD18 (endogenous plus 2XMyc-tagged DRBD18) are shown on the right. (*Bottom*) qRT-PCR analysis of RNA isolated from uninduced (−Doxy) and induced (+Doxy) DRBD18 overexpression cells using the same sets of primers as described in *A*.

To determine whether DRBD18 plays a functional role in RNA editing, we performed qRT-PCR analysis of several mitochondrial transcripts using primers that target total, preedited, or edited mRNA in a DRBD18 knockdown cell line (Fig. 2A, top; Lott et al. 2015). Our analysis revealed a significant reduction in the levels of edited A6 and COIII mitochondrial transcripts in the DRBD18 depleted cells compared to DRBD18 replete cells, with no change in the corresponding total or preedited mRNA, suggesting a role in editing or stability of these mRNAs (Fig. 2A, bottom). We observed a modest decrease in all versions of RPS12 mRNA and a decrease in total CYb mRNA upon DRBD18 knockdown, consistent with a role for DRBD18 in the stabilization of these transcripts (Fig. 2A). To further examine the involvement of DRBD18 in the editing of A6 and COIII transcripts, we generated cells expressing DRBD18 with an N-terminal 2XMyc tag, resulting in total DRBD18 levels of approximately fourfold over endogenous levels (Fig. 2B, top). qRT-PCR analysis in this overexpression cell line showed an almost twofold increase in edited A6 and COIII transcripts, confirming a specific impact of DRBD18 on these mRNAs (Fig. 2B, bottom). An increase in edited COII mRNA was also observed in DRBD18 overexpressors (Fig. 2B, bottom). We note that changes in edited mRNA levels are not always accompanied by corresponding changes in preedited mRNA levels, even in knockdowns of editing catalytic proteins (Carnes et al. 2005; McDermott et al. 2015, 2019). Thus, together with the above demonstrated DRBD18–RESC interaction, these results are consistent with a role for DRBD18 in facilitating the editing of A6 and COIII mitochondrial transcripts.

**FIGURE 3.**
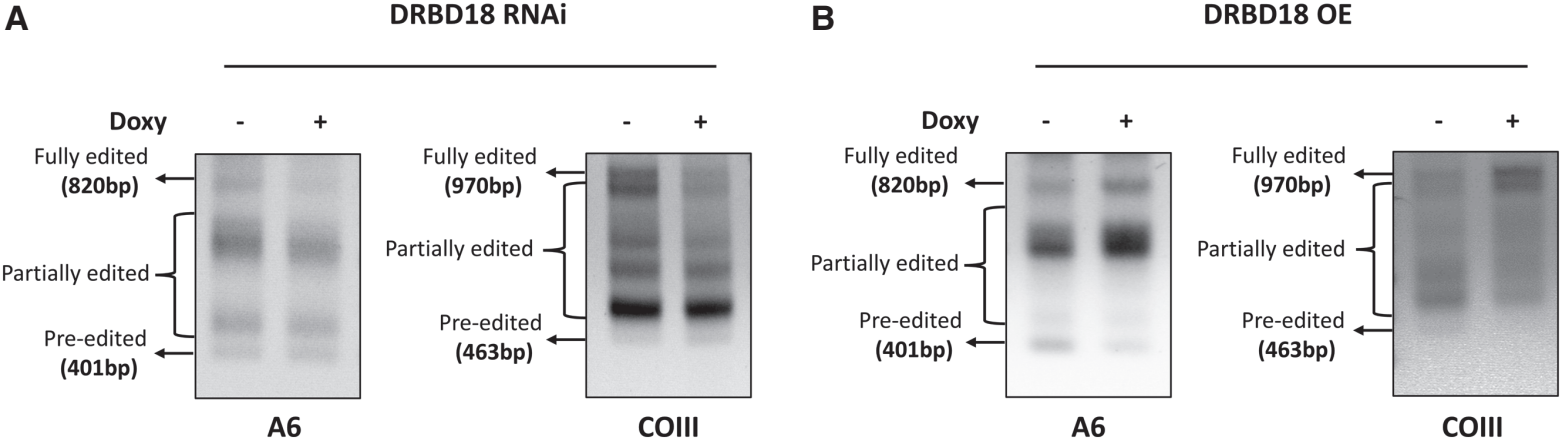
Full gene PCR analysis of A6 and COIII mRNA editing upon DRBD18 depletion and overexpression. (*A*) Agarose gel analysis of A6 and COIII RT-PCR reactions using RNA isolated from DRBD18 RNAi cells that were grown in the absence or presence of doxycycline (Doxy) for 20 h. Reverse transcription was carried out with an oligo(dT) primer that primes on the poly(A) tails of mitochondrial RNAs. Subsequent PCR was done with primers specific to the 5′ and 3′ ends of the A6 and COIII mRNAs to amplify the entire population of mRNAs including preedited, partially edited, and fully edited. (*B*) Agarose gel analysis of A6 and COIII RT-PCR reactions using RNAs isolated from DRBD18 overexpression cells that were grown in the absence or presence of doxycycline for 36 h. The reaction was performed as described in *A*.

To further explore the impact of DRBD18 on the editing of A6 and COIII transcripts, we performed gene-specific RT-PCR using primers targeting the never edited 5′ and 3′ regions of these mRNAs (Shaw et al. 2015). This assay amplifies the entire population of a given mRNA, regardless of editing status. Thus, it allows assessment of relative amounts of differentially edited mRNAs, including the large and heterogenous population of partially edited mRNAs that are not detected by qRT-PCR. Since uridine insertion occurs more frequently than uridine deletion, increased editing is detected in this assay by the presence of larger amplicons during gel electrophoresis. We found that upon RNAi of DRBD18, the levels of fully and partially edited A6 and COIII mRNA decreased compared to uninduced samples (Fig. 3A). We observed no corresponding increase in preedited A6 mRNA, mirroring qRT-PCR results, and a slight increase in preedited COIII mRNA (Fig. 3A). We next asked whether DRBD18 overexpression impacted the abundance of distinct A6 and COIII mRNA populations. Upon DRBD18 overexpression, preedited mRNA levels for both transcripts were decreased (Fig. 3B), and partially and fully edited mRNA species accumulated. While we cannot rule out additional effects of DRBD18 on edited mRNA stability, these data strongly support a role for DRBD18 in promoting the editing of A6 and COIII mRNA.

### DRBD18 associates with A6 and COIII mitochondrial transcripts

**FIGURE 4.**
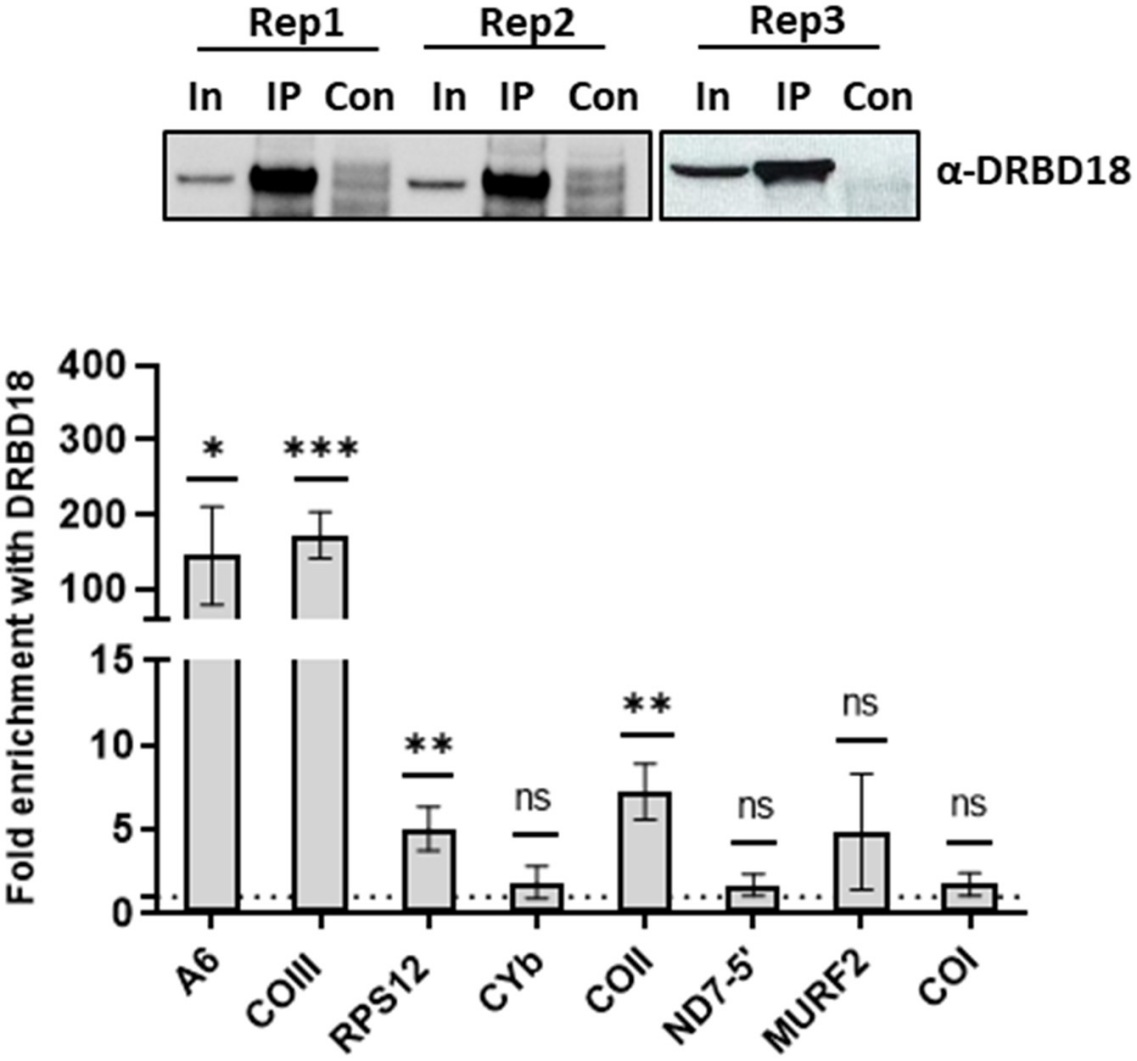
DRBD18 is associated with a subset of mitochondrial transcripts. (*Top*) Western blot showing degree of DRBD18 enrichment in three biological replicate α-DRBD18 IPs; empty beads served as the control (Con). In, input. Eight mitochondrial transcripts were quantified in DRBD18 IPs using qRT-PCR primers that amplify the total mRNA population. Values shown are fold enrichment in the DRBD18 pulldown compared to the empty bead control. The experiment was performed in three biological replicates, each with three technical qRT-PCR triplicates, and significance was calculated by Student's *t*-test. (ns) Not significant; (*) *P* < 0.05; (**) *P* < 0.01, (***) *P* < 0.001.

DRBD18 knockdown results in the upregulation of numerous cytoplasmic and nuclear RBPs (Lott et al. 2015; Bard et al. 2024). Thus, the observed impacts on RNA editing could be due to secondary effects of DRBD18 depletion. To provide evidence of a direct role for DRBD18 in editing of A6 and COIII mRNAs, we next asked whether DRBD18 associates with these mRNAs in a specific manner. To this end, we conducted biological triplicate RIP experiments followed by qRT-PCR analysis of several mitochondrial transcripts in PF *T. brucei*. IP was performed with α-DRBD18 antibodies, and qRT-PCR was performed with primers that detect nearly the entire population of a given mRNA, regardless of editing status (total RNA). Our results showed that A6 and COIII transcripts are enriched >100-fold in IPs with α-DRBD18 antibody-coated beads compared to control beads (Fig. 4). Additionally, we observed a modest but significant enrichment of RPS12 and COII transcripts, in line with the effects of DRBD18 knockdown or overexpression on these transcripts presented in Figure 2. Three other edited transcripts (CYb, ND7-5′, and MURF2) and one never-edited transcript (COI) exhibited no enrichment. Thus, DRBD18 associates with a subset of mitochondrial mRNAs, in particular A6 and COIII, either by direct RNA binding or as a component of a DRBD18 containing ribonucleoprotein. These data support a model in which DRBD18 plays a direct role in the editing of these specific transcripts.

### Methyl mutants of DRBD18 impact mitochondrial mRNA editing

**FIGURE 5.**
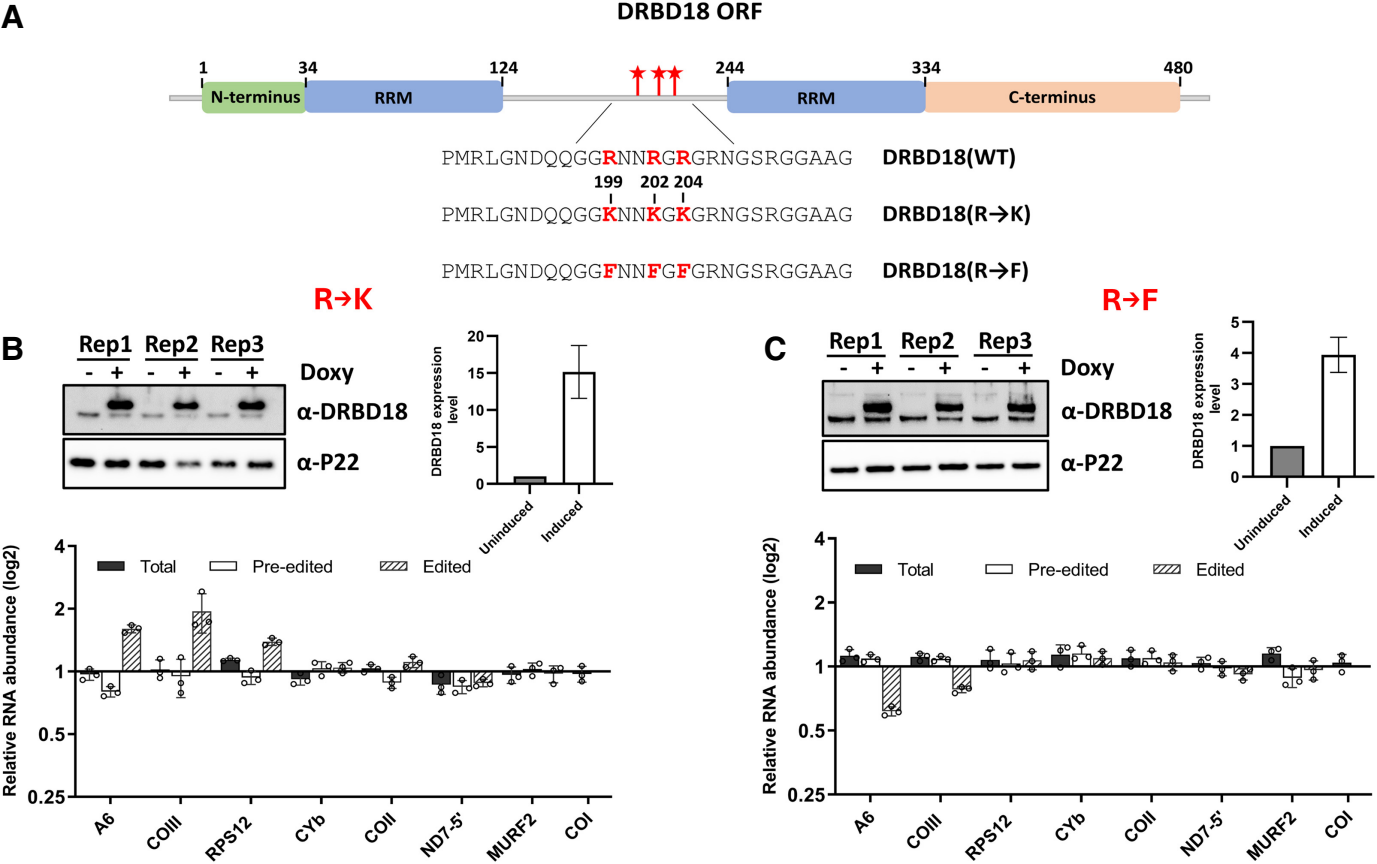
Effect of overexpression of DRBD18 methyl mutants on RNA editing. (*A*) Cartoon depicting DRBD18 domains and sites of methylation and mutations. (RRM) RNA recognition motif. Numbers indicate amino acid residues. Stars indicate sites of methylated arginines. (*B*, *top*) Western blot showing degree of 2XMyc-DRBD18 overexpression in three biological replicates of uninduced (−Doxy) and induced (+Doxy) DRBD18(R→K) cells. Levels of total DRBD18 are shown on the *right*. (*Bottom*) qRT-PCR analysis of RNA isolated from uninduced (−Doxy) and induced (+Doxy) DRBD18(R→K) overexpression cells using primer sets designed to detect total, preedited, and edited mRNA of a subset of mitochondrial transcripts. The relative abundance of each transcript in induced versus uninduced RNAi cells is shown, and the RNA levels were normalized to 18S rRNA. Three biological replicates, each with three technical replicates, were performed. (*C*) As in *B* using DRBD18(R→F) cells.

We previously reported that DRBD18 is methylated on three arginine residues that lie between its two RNA-binding domains (Fig. 5A; Lott et al. 2015). Complementation of a DRBD18 knockdown with hypomethylated or methylmimic DRBD18 differentially affected the stabilities of cytoplasmic mRNAs (Lott et al. 2015). Having demonstrated that DRBD18 affects the editing of A6 and COIII mitochondrial transcripts, we next aimed to determine how arginine methylation impacts the protein's editing function. For this, we created two doxycycline-inducible methylation mutants of DRBD18. In the first mutant, DRBD18(R→K), the three methylated arginines were replaced with lysines, resulting in a hypomethylated version of DRBD18 while maintaining the positive charge (Fig. 5A). In the second mutant, DRBD18(R→F), the methylated arginines were substituted with phenylalanine, which has been shown to mimic methylarginine (Fig. 5A; Dillon et al. 2013; Kim et al. 2015; Lott et al. 2015; Lorton and Shechter 2019). We generated cell lines overexpressing either DRBD18(R→K) or DRBD18(R→F), each tagged with N-terminal 2XMyc, for comparison to the previously discussed DRBD18(WT) overexpressor (Fig. 2B). Western blot analysis showed that DRBD18(WT) and DRBD18(R→F) are overexpressed to similar levels, while DRBD18(R→K) levels are somewhat higher (Figs. 2B and 5B,C). In some cases, we observed a modest decrease in the abundance of endogenous DRBD18, indicating regulation of total DRBD18 abundance, likely at the protein level. Overexpression of all DRBD18 variants led to a similar slight growth defect (Supplemental Fig. S1). To determine whether overexpression of methyl mutant DRBD18 supports an increase in edited A6 and COIII mRNA levels similar to that observed with DRBD18(WT) overexpression, we isolated RNA from the cell lines overexpressing the methylation mutants and performed qRT-PCR analysis on several mitochondrial transcripts. Overexpression of hypomethylated DRBD18(R→K) led to increased edited A6 and COIII levels nearly comparable to that observed with DRBD18(WT), despite their differing expression levels (compare Figs. 2B and 5B), along with an increase in edited RPS12 mRNA. In contrast, overexpression of DRBD18(R→F) resulted in a reduction in the levels of edited A6 and COIII transcripts (Fig. 5C), suggestive of a dominant negative effect on the editing of these mRNAs. Overall, these mutagenesis analyses strongly suggest that methylation of DRBD18 hinders its positive role in RNA editing.

### DRBD18 methyl mutants impact its interaction with RNA

**FIGURE 6.**
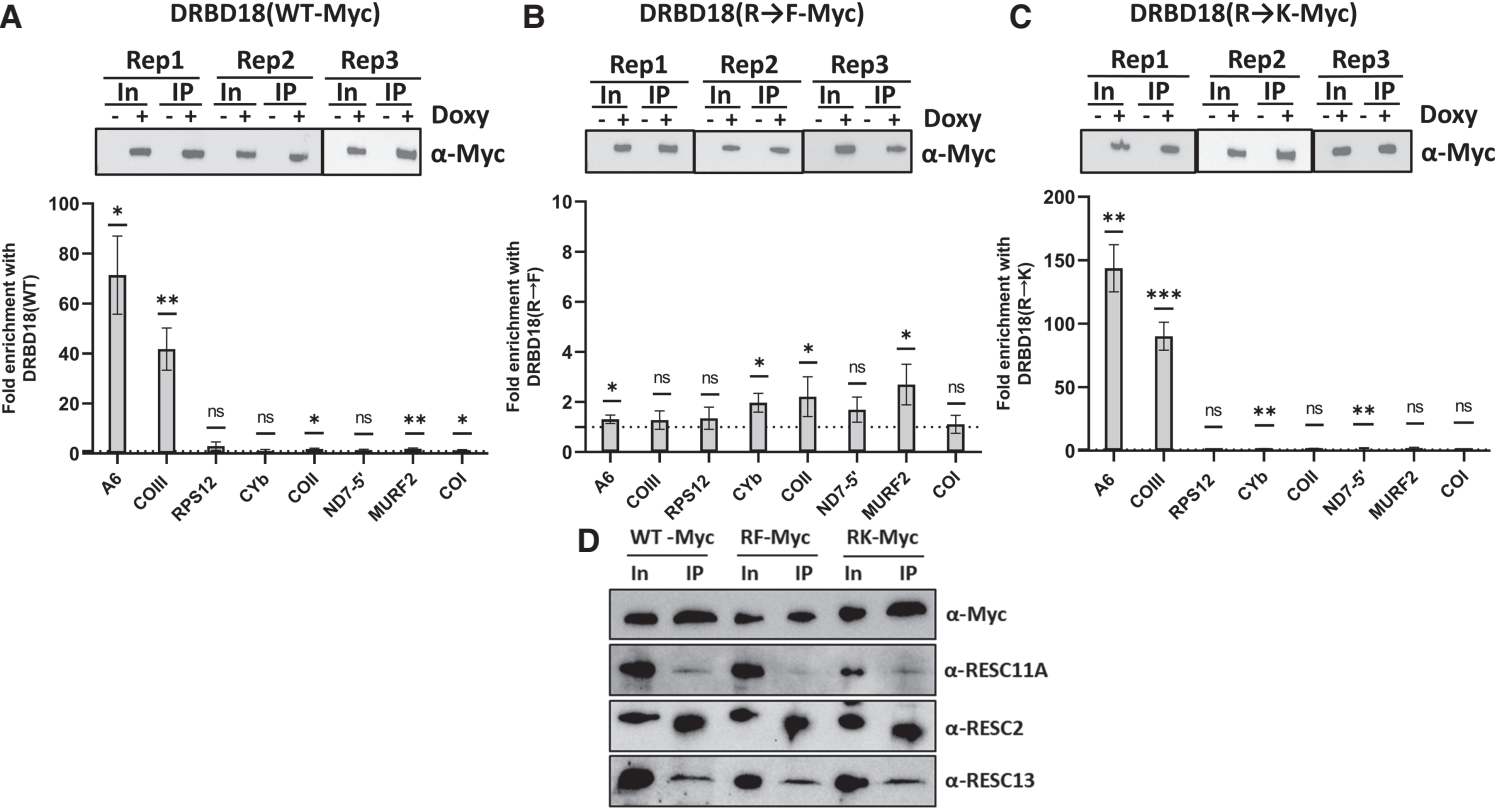
Association of wild type and methyl mutant DRBD18 with mitochondrial transcripts and RESC proteins. (*A*, *top*) Western blot showing comparable DRBD18(WT) IP in three biological replicates. In, input. (*Bottom*) Eight mitochondrial transcripts were quantified using qRT-PCR primers that amplify the total mRNA population in α-Myc IPs. Values shown are fold enrichment of a given RNA in the doxycycline-induced DRBD18(WT)-Myc IP with α-Myc antibodies compared to an α-Myc IP of uninduced cells. The experiment was performed in three biological replicates, each with three technical qRT-PCR triplicates, and significance was calculated by Student's *t*-test. (ns) Not significant; (*) *P* < 0.05; (**) *P* < 0.01, (***) *P* < 0.001. (*B*) As in *A*, with DRBD18(R→F) methylmimic variant overexpressing cells. (*C*) As in *A*, with DRBD18(R→K) hypomethyl variant overexpressing cells. (*D*) Interaction of DRBD18 overexpression variants with RESC factors. Myc-tagged DRBD18 overexpression variants (WT, R→F, and R→K) were immunoprecipitated using α-Myc antibody beads, and bound proteins were analyzed by western blot with native antibodies against RESC factors. The Myc (DRBD18), RESC11A, and RESC13 lanes were loaded 3000× input; RESC2 lanes were loaded 800× input.

Having shown the differential effects of DRBD18 methyl mutants on the editing of A6 and COIII mitochondrial transcripts, we next sought to understand the underlying mechanism. Specifically, we wanted to determine whether these hypomethyl and methylmimic mutations alter the association of DRBD18 with mitochondrial mRNAs or whether they impact the interaction between RESC factors and DRBD18. To analyze DRBD18 mRNA binding, we performed RIP experiments using an α-Myc antibody followed by qRT-PCR analysis of several mitochondrial transcripts in cell lines overexpressing Myc-tagged DRBD18(WT), DRBD18(R→K), or DRBD18(R→F). Expression of DRBD18 variants was induced with doxycycline (Fig. 6, top), and transcript levels in induced α-Myc RIPs were compared to those in the corresponding uninduced samples. Similar to endogenous DRBD18 (Fig. 4), DRBD18(WT) strongly and specifically enriched total A6 and COIII mRNA populations, with little to no binding of other tested mRNAs (Fig. 6A). Conversely, we observed little to no enrichment of any mRNA tested with the methylmimic mutant, DRBD18(R→F) (Fig. 6B). Interestingly, hypomethylated DRBD18(R→K), which supported A6 and COIII editing, enriched these two RNAs to an even greater degree than did DRBD18(WT) (Fig. 6C). These data suggest that arginine methylation of DRBD18 negatively impacts its ability to bind mitochondrial mRNAs.

We next asked whether the methylation status of DRBD18 also affects its interaction with RESC factors. We conducted co-IP experiments with Myc-tagged DRBD18 methyl variants using α-Myc beads and developed the blots against RESC11A, RESC2, and RESC13 with their native antibodies. We found that all DRBD18 variants (WT, R→F, and R→K) showed similar interactions with the tested RESC factors (RESC11A, RESC2, RESC13) (Fig. 6D). Because the interaction between DRBD18 and RESC factors is RNase-sensitive (Fig. 1A), it was surprising that these protein–protein interactions persisted in the absence of RNA binding for the methylmimic mutant (compare Fig. 6B and 6D). While it is possible that the RNAs mediating the interaction are not among those mRNAs tested by RIP, the continued ability of DRBD18(R→F) to interact with RESC factors despite its lack of binding to numerous mitochondrial mRNAs suggests that it associates with RESC in a manner distinct from that of WT DRBD18. Both altered RESC binding and lack of RNA interaction likely contribute to the inability of methylmimic DRBD18(R→F) to promote editing of A6 and COIII mRNAs.

## DISCUSSION

Several mass spectrometry studies identified interactions between DRBD18 and RESC factors, suggesting a role of this protein in mitochondrial U-indel RNA editing in addition to its previously reported impacts on nuclear-encoded mRNAs (Lott et al. 2015; Bishola Tshitenge et al. 2022; Liu et al. 2023). In this study, we confirm DRBD18–RESC interactions, explore the functional significance of DRBD18 in RNA editing, and examine how arginine methylation affects this process. Overall, we conclude that DRBD18 directly impacts the editing of A6 and COIII mRNAs, and that this function is coordinated by DRBD18's arginine methylation status. This is the first study to implicate DRBD18 as a U-indel RNA editing auxiliary factor.

The effect of DRBD18 on editing was specific to A6 and COIII mRNAs, with opposing effects on both mRNAs upon DRBD18 knockdown and overexpression (Fig. 2). These data add DRBD18 to the growing list of editing auxiliary factors shown to promote editing of A6 mRNA (Shaw et al. 2015; Carnes et al. 2023; Dubey et al. 2023). We note that A6 and COIII mRNAs are among the largest and most extensively edited mitochondrial mRNAs, with fully edited sizes of 820 and 970 nt, respectively. Longer mRNAs likely contribute to more complex mRNA–mRNA and gRNA–mRNA structures than are present with shorter edited mRNAs. Thus, longer mRNAs may require more auxiliary factors to help facilitate their editing through modulation or resolution of these structures.

In addition to its role in RNA editing, DRBD18 may also impact the stability of RPS12 and CYb mRNAs as both total mRNA populations were decreased upon DRBD18 RNAi, as was preedited RPS12 (Fig. 2). Such an effect is analogous to the reported role of DRBD18 in regulating stability of nuclear-encoded mRNAs (Lott et al. 2015). A recent quantitative mass spectrometry study identified interactions not only between DRBD18 and RESC, but also between DRBD18 and both components of the 5′ pyrophosphate processome (PPsome), as well as the KPAF1 component of the kinetoplast polyadenylation complex (KPAC) (Bishola Tshitenge et al. 2022). The KPAC adds a stabilizing poly(A) tail to the 3′ termini of mRNAs, while the PPsome acts as a “protein cap” at the 5′ termini that stabilizes monophosphorylated mRNAs by circularizing the mRNA and interacting with KPAC at the 3′ termini (Sement et al. 2018; Mesitov et al. 2019; Aphasizheva et al. 2020a). These reported interactions between DRBD18 and mitochondrial mRNA stabilizing proteins implicate DRBD18 in the regulation of mitochondrial mRNA stability and hint at possible mechanisms.

DRBD18 is an abundant RBP that reportedly localizes to both the cytoplasm and nucleus, and regulates expression of many nuclear-encoded proteins (Lott et al. 2015; Mishra et al. 2021; Bishola Tshitenge et al. 2022; Ciganda et al. 2023; Bard et al. 2024). As DRBD18 localization to mitochondria has not been described, our results pose the question of whether DRBD18's impact on mitochondrial RNA editing is direct or a secondary effect of its knockdown and overexpression. While most mitochondrially localized proteins contain cleavable amino terminal import sequences (Sunter et al. 2023), the DRBD18 amino acid sequence does not contain a recognizable mitochondrial import sequence at its N-terminus, nor is it predicted to be mitochondrially localized by TargetP or Psort algorithms. Indeed, our experiments comparing WT, methylmimic, and hypomethylated DRBD18 were conducted with proteins harboring 2XMyc tags at their amino termini, which is expected to block the import of mitochondrial proteins with conventional amino terminal import sequences. Nevertheless, internal mitochondrial targeting signals have been reported in *T. brucei* (Priest and Hajduk 2003; Shikha and Schneider 2020; Singha et al. 2021; Darden et al. 2024). Moreover, several lines of evidence suggest that some fraction of cellular DRBD18 is present in the mitochondria and does indeed directly impact editing. Firstly, 20% of proteins specifically associated with DRBD18 compared to a GFP control through quantitative mass spectrometry are mitochondrial proteins (Bishola Tshitenge et al. 2022). It is unlikely that each one of those protein interactions are nonspecific or also occur outside of the mitochondria. Secondly, when analyzing the association of DRBD18 with the editing machinery, we found that DRBD18 specifically interacts with RESC factors and not with RECC, REH2C, or editing auxiliary factors (Fig. 1). This confirms that DRBD18 is not randomly interacting with mitochondrial proteins after cell lysis, but rather engaging in specific interactions. Thirdly, using RIP analysis, we determined that DRBD18 specifically interacts with A6 and COIII mitochondrial mRNAs (Figs. 4, 6A and 6C), the very RNAs whose editing it impacts (Fig. 1). Since our RIP analysis entails UV-crosslinking prior to cell lysis as well as stringent washing using a high salt concentration, this further indicates that DRBD18-A6/COIII mRNA interactions occur in vivo. Taken together, our data strongly support a direct role of DRBD18 in A6 and COIII RNA editing, indicating that some mitochondrial import of DRBD18 occurs. The mechanism by which a small fraction of DRBD18 is imported into mitochondria will be of future interest.

We previously reported that arginine methylation of DRBD18 impacts its function, with hypomethylated and methylmimic variants differentially affecting the stability of distinct mRNAs (Lott et al. 2015). We show here that only overexpression of hypomethylated DRBD18 supports increased editing of A6 and COIII mRNAs. Conversely, overexpression of methylmimic DRBD18 specifically hinders editing of these mRNAs (Fig. 5). The effect of methylation appears to be driven largely by modulation of DRBD18's RNA-binding activity. Whereas WT and hypomethylated DRBD18 exhibit robust association with A6 and COIII mRNAs, methylmimic DRBD18 fails to bind these transcripts (Fig. 6). Arginine methylation has been shown to inhibit or reduce protein–RNA binding in other systems, including the cellular nucleic acid-binding protein in humans (Wei et al. 2014), the fragile X mental retardation protein (Stetler et al. 2006), and the Tat protein of human immunodeficiency virus (Xie et al. 2007). In *T. brucei* mitochondria, TbPRMT1-mediated methylation of KRBP16 decreases its association with gRNA while enhancing its association with mRNA (Goulah et al. 2007). In contrast to RNA binding, WT, hypomethylated, and methylmimic DRBD18 associate to a similar degree with RESC proteins, at least those factors tested here (Fig. 6D). This result was surprising since DRBD18–RESC interactions are RNase-sensitive (Fig. 1A), and it suggests that methylmimic DRBD18 binds RESC in a manner distinct from that of hypomethylated DRBD18. It is possible that the inability of methylmimic DRBD18 to bind A6 and COIII mRNAs is not a direct effect but is a consequence of an altered DRBD18–RESC interaction that results in decreased mRNA association and hindered editing progression. Of interest is the question of whether methylation of DRBD18 plays a regulatory role in U-indel editing. While no arginine demethylases have been definitely described in any system (Blanc and Richard 2017), it is plausible that DRBD18 methylation is increased under conditions requiring reduced levels of edited A6 or COIII mRNAs. Such regulation would likely be catalyzed by protein arginine methytransferases (PRMTs) within the mitochondria. While this localization has not been confirmed for any of the four *T. brucei* PRMTs (Pelletier et al. 2005; Pasternack et al. 2007; Fisk et al. 2009b, 2010; Fisk and Read 2011), we previously identified 167 arginine-methylated proteins with diverse functions localized in the mitochondria (Fisk et al. 2013). The abundance of arginine-methylated proteins in the mitochondria strongly suggests that some substrates undergo modification within this compartment. Furthermore, our data support the presence of both methylated and unmethylated forms of DRBD18 in the mitochondria. We showed that hypomethylated DRBD18 binds substantially greater amounts of A6 and COIII mRNAs than does the WT protein (compare Fig. 6A and 6C), suggesting that the WT population consists of both unmethylated protein that does bind A6 and COIII mRNA and methylated protein that is nearly incapable of binding these mRNAs. Alterations to the balance of methylated versus unmethylated DRBD18 would add a new layer of regulation to the U-indel RNA editing process.

## MATERIALS AND METHODS

### Cell line generation and growth conditions

PF *T. brucei* strain 29-13 (Wirtz et al. 1999) and all the other cell lines derived from this strain were grown at 27°C in SM medium (Cunningham 1977) supplemented with 10% fetal bovine serum and containing hygromycin (50 µg/mL) and G418 (15 µg/mL). The 29-13 derivative of PF cells harboring the doxycycline-inducible DRBD18 (Tb927.11.14090) RNAi constructs described earlier (Lott et al. 2015) were grown with the addition of phleomycin (2.5 µg/mL). To induce the DRBD18 RNAi, the cell line was incubated for 20 h with 1 µg/mL doxycycline.

Cell lines expressing N-terminally 2XMyc-tagged DRBD18 were created by amplifying the DRBD18 ORF using DRBD18 5′ HindIII For (CCCAAGCTTGAACAAAAACTCATCTCAGAAGAGGATCTGGAACAAAAACTCATCTCAGAAGAGGATCTGATGCAAGGCGCATACGGA) and DRBD18 3′ Rev BamHI (CCCGGGGGATCCTTATGCTGAACCATTTTCC) primers. The amplified product was ligated into the HindIII/BamHI sites of pLEW100 (Wirtz et al. 1999) containing a 2XMyc N-terminal tag using Infusion cloning. Triple R-to-K and triple R-to-F mutant constructs were generated as described previously (Lott et al. 2015) through KOD polymerase (Sigma) mutagenesis and verified by DNA sequencing. The pLEW100_DRBD18 (WT), pLEW100_DRBD18(R-K), and pLEW100_DRBD18(R-F) plasmids were digested with NotI, purified, and transfected into the *T. brucei* 29-13 background. Cells harboring pLEW100-based plasmids were selected with puromycin (1 µg/mL), and clones were obtained by limiting dilution. Growth rates were analyzed for three biological replicates of each cell line.

### Co-IP and western blot

Approximately 1 × 10^10^ *T. brucei* 29-13 cells were harvested and washed with PBS, and mitochondria were enriched as described previously (Hashimi et al. 2008; McAdams et al. 2018). Enriched mitochondria were lysed in N150 buffer (50 mM Tris [pH 8], 150 mM NaCl, 0.1% [v/v] NP-40, and 5 mM β-ME) with 1% (v/v) Triton X-100 in the presence of cOmplete Protease Inhibitor Cocktail (Roche). Lysate was divided into two fractions: one was incubated with 200 U SUPERase-In RNase Inhibitor (Invitrogen) and 0.5 μg/mL DNase I, while the other was incubated with DNase I and a nuclease cocktail containing 60 μg RNase A, 2500 U RNase T1 (Ambion), 28 U RNase H (Invitrogen), and 2040 U micrococcal nuclease for 1 h on ice. Lysates were then incubated with α-DRBD18 antibodies (Lott et al. 2015; Ciganda et al. 2023) attached to protein A fast-flow beads (GE Healthcare) for 2 h at 4°C. After washing with wash buffer (50 mM Tris [pH 8], 300 mM NaCl, 0.1% [v/v] NP-40, and 5 mM β-ME), the bound proteins were electrophoresed on a 12% SDS-PAGE gel and transferred to a nitrocellulose membrane. Membranes were blocked with 5% nonfat dry milk in Tris-buffered saline with Tween-20 (TBST) and probed with polyclonal antibodies against DRBD18 (Lott et al. 2015), RESC2 (Ammerman et al. 2011), RESC8 (McAdams et al. 2019), RESC10 (Dubey et al. 2021), RESC11A (Simpson et al. 2017), RESC13 (Fisk et al. 2008), RESC14 (McAdams et al. 2018), KRBP72 (Dubey et al. 2024), KH2F2 (Kumar et al. 2016), MRP1 (Fisk et al. 2009a), MRP2 (Fisk et al. 2009a), P22 (Hayman et al. 2001), RBP16 (Ammerman et al. 2008), TbRGG1 (Fisk et al. 2009a), and RECC complex (KREPA1, KREPA2, KREPA3, and KREL1) (Panigrahi et al. 2001). Blots were washed with TBST buffer and subsequently probed with secondary antibodies, either goat α-rabbit HRP (1:2000 dilution) or goat α-mouse HRP (1:2000). Signals were detected using an ECL preparation as recommended by the manufacturer (Thermo Fisher Scientific), visualized on a Chemi Doc imaging system (Bio-Rad).

For pulldown in the reverse direction, 1 × 10^10^ cells harboring endogenously tagged RESC13-MHT, RESC14-MHT, or RESC2-PTP (Wackowski et al. 2024) were harvested and lysed in N150 buffer with 1% (v/v) Triton X-100 in the presence of cOmplete Protease Inhibitor Cocktail (Roche), 1 mM CaCl_2_, and 0.5 μg/mL DNase I. Lysate was then divided into two fractions: one fraction was incubated with 200 U SUPERase-In RNase Inhibitor (Invitrogen) and 0.5 μg/mL DNase I, whereas the other fraction was incubated with DNase I and a nuclease cocktail containing 60 μg RNase A, 2500 U RNase T1 (Ambion), 28 U RNase H (Invitrogen), and 2040 U micrococcal nuclease for 1 h on ice. The two lysates were then incubated with IgG Sepharose 6 Fast Flow beads (Cytiva) for 2 h on ice. Beads were washed with N150 buffer, and for MHT-RESC13 and MHT-RESC2, they were incubated in TEV cleavage buffer (10 mM Tris [pH 8.0], 150 mM NaCl, 0.1% [v/v] NP-40, 0.5 mM EDTA, 1 mM DTT) with 100 U AcTEV Protease (Invitrogen) at 4°C overnight. For MHT-RESC14, glycine (100 mM [pH 3.0]) elution, followed by neutralization with 1 mM Tris-HCl (pH 8.0), was performed. IP was performed as described previously (McAdams et al. 2018; Wackowski et al. 2024). TEV elutions (RESC13 and RESC2) and glycine elution (RESC14) were subjected to western blot analysis to detect the target protein using either native antibodies (RESC2 and RESC13) or α-Myc antibodies (Invitrogen; 1:2000 dilution) (RESC14), and interacting partner DRBD18 was detected using α-DRBD18 antibody.

For pulldown of DRBD18 overexpression variants, 1 × 10^10^ cells were harvested, and then washed in 1× PBS. Cell lysates were precleared using Superdex 200 beads (Cytiva). IP of 2XMyc-tagged DRBD18 variants from cleared lysate was performed using α-Myc antibody as described previously (McAdams et al. 2018). Glycine elutions were subjected to western blot analysis to detect the DRBD18 variants using α-Myc antibody and interacting RESC factors (RESC11A, RESC2, and RESC13) using their native antibodies.

### qRT-PCR analysis

RNA was isolated from DRBD18 RNAi cells grown in the presence or absence of 1 µg/mL doxycyxcline for 20 h. Cells overexpressing DRBD18(WT) or methyl mutant variants were grown in the presence or absence of 1 µg/mL doxycyxcline for 36 h. To normalize overexpression levels to those in the other cells lines, DRBD18(R→F) cells were grown in the presence or absence of 10 µg/mL doxycycline 36 h. RNA was isolated using TRIzol reagent (Ambion) and treated with DNase I (Ambion), according to the manufacturer's instructions. RNA was extracted using phenol/chloroform followed by ethanol precipitation. The purity of the RNA was measured using NanoDrop 1000, and the 260/280 ratio was ∼2, and quality of the RNA was analyzed on a 1.5% of TBE agarose gel. RNA (1 μg) was reverse transcribed using random hexamer primers and the iScript Reverse Transcriptase kit (Bio-Rad). qRT-PCR was performed using *T. brucei* established primers to detect levels of preedited, edited, and total mitochondrial transcripts, with normalization to 18S rRNA (Carnes et al. 2005; McAdams et al. 2019; Dubey et al. 2023).

### RT-PCR analysis of A6 and COIII transcripts

RNA was extracted from uninduced and doxycycline-induced DRBD18 RNAi and DRBD18 overexpression cells. Oligo(dT) primed cDNA was prepared by reverse transcription with iScript Reverse Transcriptase kit (Bio-Rad). A6 and COIII specific primers, which anneal to the never-edited 5′ and 3′ regions that flank the edited region of both A6 and COIII mRNA, were used for RT-PCR (Shaw et al. 2015).

### RIP/qRT-PCR of endogenous DRBD18 and its overexpression variants

PF *T. brucei* 29-13 cells, 2XMyc-tagged DRBD18(WT) overexpression cells, and cells overexpressing DRBD18(R→F) or DRBD18(R→K) were harvested (1 × 10^10^ cells each), washed once with cold 1× PBS (pH 7.4). Cells were resuspended in 25 mL no FBS SM media to a concentration of ∼5 × 10^9^ cells/mL^−1^ and transferred to a 100 × 15 mm Petri dish. Plates were incubated on ice, and UV irradiated at 400 mJ/cm^2^ in a Stratalinker 1800 (Stratagene). Cells were pelleted, washed with PBS, snap-frozen in liquid N_2_, and stored at −80°C until use. Cells were resuspended in 10 mL of lysis buffer (Tris-HCl [pH 7.5], 20 mM NaCl, 0.1% NP40, and 1% Triton X-100) and then lysed by passing through a 21-gauge needle 20 times. Cell lysate was centrifuged at 18,000 rpm for 30 min at 4°C, and the supernatant was adjusted to 150 mM NaCl. RIP was performed as described previously (McAdams et al. 2018, 2019; Ciganda et al. 2023). Briefly, for endogenous DRBD18, the cell lysate was immunoprecipitated with α-DRBD18 antibodies (Lott et al. 2015) attached to protein A fast flow beads (GE Healthcare); crosslinked protein A fast flow beads without antibody were used as the control. For 2XMyc-tagged DRBD18 and its methyl mutants, Myc beads (ICL) were used for IP. Captured protein–RNA complexes were washed in Tris-HCl (pH 7.5), 300 mM NaCl, 0.1% NP40, and 5% of the beads from each sample were used for western blot to confirm the pulldown of DRBD18. The supernatant was removed after DNase I (Sigma) treatment followed by proteinase K (Roche) treatment. RNA was extracted with phenol/chloroform followed by ethanol precipitation. RNA was DNase-treated (Ambion DNA-free DNase kit), and 500 ng of RNA was converted to cDNA with *T. brucei* established gene-specific primers targeting the total mRNA (A6, COIII, RPS12, CYb, COII, ND7 5′, MURF2, and COI) and 18S rRNA using the iScript cDNA synthesis kit (Bio-Rad). cDNA was amplified using SsoAdvanced PreAmp Supermix (Bio-Rad) and then used for qRT-PCR, with 18S rRNA used for normalization. The ΔΔ*Ct* method was used to determine the fold change as described previously (McAdams et al. 2018).

## SUPPLEMENTAL MATERIAL

Supplemental material is available for this article.

## Figures and Tables

**Parul Pandey:**
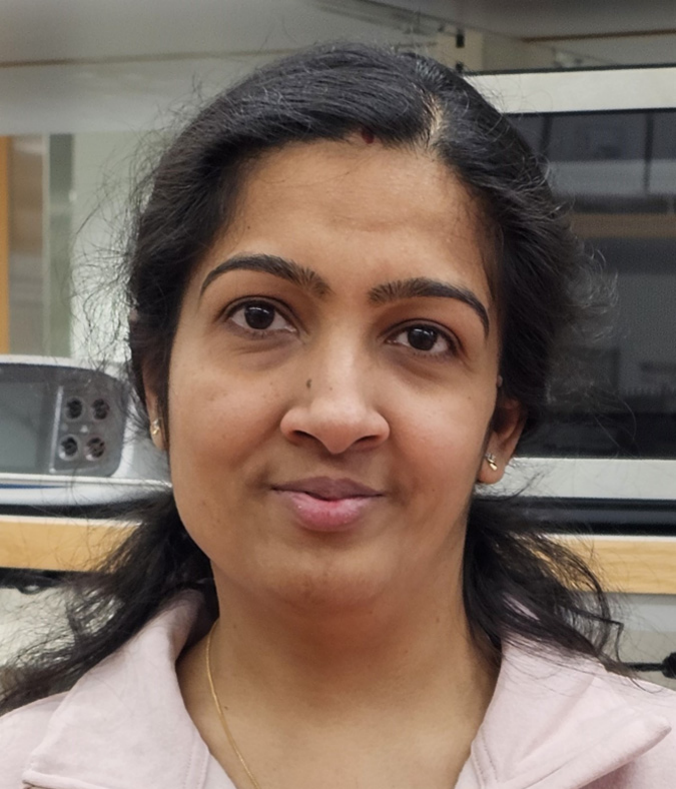


## MEET THE FIRST AUTHOR

***Meet the First Author(s)* is an editorial feature within *RNA*, in which the first author(s) of research-based papers in each issue have the opportunity to introduce themselves and their work to readers of *RNA* and the RNA research community. Parul Pandey is the first author of this paper, “DRBD18 acts as a transcript-specific RNA editing auxiliary factor in *Trypanosoma brucei*.” Parul is a postdoctoral fellow in the Read Lab at the University at Buffalo. Her research focuses on the posttranslational modification of RNA-binding protein DRBD18 by arginine methylation that modulates RNA processing, stability, translation and mitochondrial mRNA editing in the parasite *T. brucei*.**

**What are the major results described in your paper and how do they impact this branch of the field?**

This work uncovers the involvement of DRBD18 in the mitochondrial mRNA editing process of the parasite *T. brucei*. Here we demonstrated the specific and RNase-sensitive interaction of DRBD18 with multiple RESC factors. Depletion of DRBD18 reduces the edited A6 and COIII mitochondrial transcripts, while its overexpression increases their levels. We also confirmed the direct involvement of DRBD18 in editing these mRNAs by RNA immunoprecipitation/qRT-PCR. Additionally, the study identifies that the hypomethylated form of DRBD18 is essential for this process, marking the first report of a mitochondrial function for DRBD18 and establishing it as a novel RNA editing auxiliary factor.

**What led you to study RNA or this aspect of RNA science?**

During my graduate research, I studied the transcription machinery of a plant growth-promoting rhizobacterium. However, I was eager to shift my focus to pathogenic microorganisms and delve deeper into RNA biology. Joining Dr. Laurie Read's lab provided the perfect opportunity to broaden my understanding of RNA biology, including posttranscriptional and posttranslational modifications, RNA editing, and RNA turnover in parasites.

**If you were able to give one piece of advice to your younger self, what would that be?**

If I could give one piece of advice to my younger self, it would be to embrace challenges and setbacks as opportunities for growth and learning. Research is a journey filled with uncertainties, and every unexpected result or obstacle is a chance to develop resilience, creativity, and a deeper understanding of science. Trust the process, remain curious, and never hesitate to ask questions or seek help from mentors and colleagues—they are invaluable resources along the way.

**Are there specific individuals or groups who have influenced your philosophy or approach to science?**

Yes, several individuals and groups have significantly shaped my philosophy and approach to science. My graduate advisor played a pivotal role in fostering my curiosity and encouraging a rigorous, methodical approach to research. Joining Dr. Read's lab has further deepened my understanding of RNA biology, inspiring me to explore complex questions with a collaborative and multidisciplinary mindset. Additionally, interactions with peers and mentors in various research settings have reinforced the importance of open communication, perseverance, and a commitment to impactful science.

**What are your subsequent near- or long-term career plans?**

In the near term, I aim to continue expanding my expertise in RNA biology and molecular parasitology through postdoctoral research, focusing on uncovering novel regulatory mechanisms that can inform therapeutic strategies. Long-term, I aspire to establish independent research where I can mentor the next generation while contributing to advancements in RNA biology and infectious disease research.
